# Cam morphology but neither acetabular dysplasia nor pincer morphology is associated with osteophytosis throughout the hip: findings from a cross-sectional study in UK Biobank

**DOI:** 10.1016/j.joca.2021.08.002

**Published:** 2021-11

**Authors:** B.G. Faber, R. Ebsim, F.R. Saunders, M. Frysz, J.S. Gregory, R.M. Aspden, N.C. Harvey, G. Davey Smith, T. Cootes, C. Lindner, J.H. Tobias

**Affiliations:** † Musculoskeletal Research Unit, University of Bristol, UK; ‡ Medical Research Council Integrative Epidemiology Unit, University of Bristol, UK; § Division of Informatics, Imaging and Data Sciences, The University of Manchester, UK; || Centre for Arthritis and Musculoskeletal Health, University of Aberdeen, UK; ¶ Medical Research Council Lifecourse Epidemiology Unit, University of Southampton, UK

**Keywords:** Cam, Pincer, Acetabular dysplasia, DXA, Osteoarthritis, Epidemiology

## Abstract

**Objectives:**

To examine whether acetabular dysplasia (AD), cam and/or pincer morphology are associated with radiographic hip osteoarthritis (rHOA) and hip pain in UK Biobank (UKB) and, if so, what distribution of osteophytes is observed.

**Design:**

Participants from UKB with a left hip dual-energy X-ray absorptiometry (DXA) scan had alpha angle (AA), lateral centre-edge angle (LCEA) and joint space narrowing (JSN) derived automatically. Cam and pincer morphology, and AD were defined using AA and LCEA. Osteophytes were measured manually and rHOA grades were calculated from JSN and osteophyte measures. Logistic regression was used to examine the relationships between these hip morphologies and rHOA, osteophytes, JSN, and hip pain.

**Results:**

6,807 individuals were selected (mean age: 62.7; 3382/3425 males/females). Cam morphology was more prevalent in males than females (15.4% and 1.8% respectively). In males, cam morphology was associated with rHOA [OR 3.20 (95% CI 2.41–4.25)], JSN [1.53 (1.24–1.88)], and acetabular [1.87 (1.48–2.36)], superior [1.94 (1.45–2.57)] and inferior [4.75 (3.44–6.57)] femoral osteophytes, and hip pain [1.48 (1.05–2.09)]. Broadly similar associations were seen in females, but with weaker statistical evidence. Neither pincer morphology nor AD showed any associations with rHOA or hip pain.

**Conclusions:**

Cam morphology was predominantly seen in males in whom it was associated with rHOA and hip pain. In males and females, cam morphology was associated with inferior femoral head osteophytes more strongly than those at the superior femoral head and acetabulum. Further studies are justified to characterise the biomechanical disturbances associated with cam morphology, underlying the observed osteophyte distribution.

## Introduction

Hip osteoarthritis (OA) is a common condition that causes considerable morbidity often leading to costly total hip replacements (THR)^1^^,^^2^. Differences in hip morphology have long been postulated as risk factors, including acetabular dysplasia (AD), and cam and pincer morphologies^3^. AD is associated with under-coverage of the acetabulum over the femoral head and is considered a consequence of milder forms of developmental dysplasia of the hip (DDH)^4^^,^^5^. Severe DDH is strongly associated with hip OA whereas AD shows inconsistent associations^5^, ^6^, ^7^. Cam morphology, which represents bulging of the lateral femoral head leading to an aspherical appearance, and pincer morphology, comprising increased coverage of the acetabulum over the femoral head, both have been suggested to cause OA via femoro-acetabular impingement (FAI). The biomechanical concept of aberrant forces due to impingement of the superolateral femoral head on the lateral acetabulum during hip movement in particular flexion, abduction and internal rotation^8^^,^^9^.

An individual's hip morphology develops through gestation, childhood and adolescence well before the onset of OA^3^^,^^10^. Genetic loci have been associated with different hip morphologies including DDH indicating a genetic predisposition^11^^,^^12^. Observational studies suggest cam morphology forms in adolescence when the metaphysis fuses, with increased physical activity implicated as a risk factor^13^^,^^14^. FAI syndrome is recognised as a cause of hip pain in younger individuals, diagnosis of which is supported by relevant examination findings and either cam and/or pincer morphologies in the absence of OA^8^^,^^15^. Several studies suggest that surgery to correct the hip morphologies implicated in FAI improves symptoms such as pain^16^, ^17^, ^18^. Conceivably, surgery to correct these hip morphologies and prevent FAI might also prove useful in reducing the risk of developing OA. However, whether FAI is a risk factor for hip OA in the general population remains unclear. Whereas cam morphology is associated with an increased risk of radiographic hip OA (rHOA) and THR^5^, pincer morphology does not appear to be a risk factor for hip OA^7^^,^^19^. FAI has been proposed to cause hip OA in patients with cam and/or pincer morphologies secondary to impingement^20^ but as yet the precise mechanism remains unclear. A systematic review showed labral deformities are associated with cam morphology but the authors concluded causality could not be inferred from the studies^21^. No population studies have explored the distribution of osteophytes in individuals with these shape morphologies, which might give some indication as to any underlying biomechanical disturbance.

In the present study, we sought to establish the importance of hip morphology as a risk factor for OA by examining whether AD, cam and/or pincer morphology are related to rHOA and/or hip pain. In particular, we aimed to determine what distributions of osteophytes, if any, are associated with these hip morphologies. We used high resolution dual-energy X-ray absorptiometry (DXA) scans of the hip (previously validated for the use of detecting rHOA^22^), from a sub-sample of UK Biobank (UKB), and applied a novel automated method for ascertaining hip morphology to address these questions.

## Materials and methods

### Population

UKB is a mixed sex cohort, based in the UK, which prospectively recruited 500,000 adults aged 40–69 years old between 2006 and 2010. The UKB Ethics Advisory Committee oversees the maintenance, development and use of UKB data and its approval covers this study. The participants underwent extensive genetic and physical phenotyping (http://biobank.ctsu.ox.ac.uk/crystal/), and consented to their data being used in this study^23^. The extended imaging study has conducted hip DXA scans (iDXA GE-Lunar, Madison, WI) on nearly 50,000 individuals to date using a standardised protocol that positioned the patient's hip in 15–25° of internal rotation^24^. The sample was weighted to include equal numbers of each sex, the first 20% of individuals selected were taken from those with a self-reported diagnosis of OA at any site, the remaining 80% were selected randomly from those with a hip DXA^25^. All demographic information was taken from measurements or questionnaires conducted on the same day as the DXA scans.

### DXA mark up, radiographic measure of osteoarthritis and hip pain

**Fig. 1:**
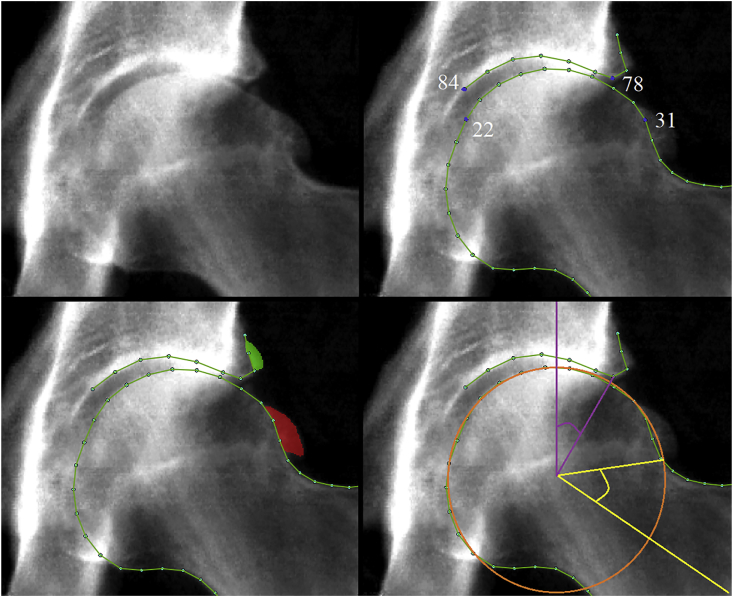
Top left image: Sample DXA scan from UKB showing rHOA. Top right image: Outline points are shown around the femoral head and acetabulum on the same DXA scan. Points 22, 31, 78 & 84 are labelled and blue, they mark the point boundaries between which mJSW is calculated. Bottom left image: Outline points are shown along with osteophyte mark-ups where green denotes acetabular osteophytes and red superior femoral osteophytes. Bottom right image: Circle of best fit is shown in orange with purple lines depicting how LCEA is calculated and yellow lines depicting how AA is calculated.

A detailed description of the DXA mark up and derivation of parameters related to rHOA is available^25^. In brief, a machine learning algorithm placed 85 outline points around the left femoral head and acetabulum^26^^,^^27^. The points were manually checked and corrected where necessary. All osteophytes were marked up using a custom tool (University of Manchester) which allows the user to shade/identify pixels where an osteophyte is visible (Fig. 1), at the lateral acetabulum, superolateral femoral head, and inferomedial femoral head. Femoral head osteophytes are referred to as superior and inferior femoral head osteophytes for simplicity. Outline points were moved to the internal boundary of an osteophyte if present (Fig. 1). Osteophyte area was used to derive osteophyte grade, based on thresholds identified from receiver operating characteristic curve (ROC) analyses comparing osteophyte area with osteophyte grade assessed semi-quantitatively in a subset of images. Superior minimum joint space width (mJSW) in millimetres (mm) was automatically measured between lines drawn through points 78–84 on the acetabulum and points 22–31 on the femoral head (Fig. 1). From mJSW semi-quantitative joint space narrowing (JSN) was calculated by applying ROC-derived thresholds to height adjusted mJSW measures, as these were more accurate (greater area under the curve) than using mJSW alone^25^. Repeatability for the presence of osteophytes intra-reader kappa of 0.80–0.91 was obtained with repeat readings of 500 images more than 2 months after initial grading and JSN on 100 images giving a kappa of 0.93. RHOA was defined as the presence of both grade ≥1 JSN and a grade ≥1 osteophyte at any location^28^^,^^29^. In addition, we employed a more stringent threshold, termed rHOA grade ≥2, requiring the presence of a grade ≥2 osteophyte and grade ≥2 JSN. Subchondral sclerosis and cysts were not examined as part of this study due to their relative infrequency^30^. A binary hip pain variable was derived from the following question: *“Have you had hip pains for more than* 3 months*?”* The question was not side-specific and the cause of hip pain is not identified.

### Alpha angle

To automatically derive alpha angle (AA), a custom Python script was developed that fits a circle of best fit using the outline points 15–28 around the femoral head^31^. The script calculates the angle between a line passing through the centre of the femoral head and neck, and a line passing through the centre of the femoral head and the point at which the femoral head–neck junction leaves the circle of best fit (Fig. 1). An in-depth description of these methods including validation experiments has previously been published^32^. Cam morphology was defined as AA ≥60°^33^^,^^34^. For repeatability, 100 images were reassessed more than 2 months after initial reading with the same methods. The AA from each assessment was compared giving a concordance correlation coefficient 0.84, and cam morphology comparison gave a kappa 0.81 (97% agreement).

### Lateral centre-edge angle

To automatically derive the lateral centre-edge angle (LCEA), a custom Python script was developed that calculates the angle between a line passing through the lateral edge of the acetabulum (defined by outline point 78) and the centre of the femoral head (defined by the circle of best fit as described above), and a line which passes perpendicular to the image *x*-axis through the centre of the femoral head (Fig. 1)^19^. Pincer morphology was defined as a LCEA of ≥45° and AD as a LCEA <25°^7^^,^^19^. 100 images were reassessed for repeatability more than 2 months after initial reading. The LCEA from each assessment was compared giving a concordance correlation coefficient 0.98, pincer morphology comparison gave a kappa 0.94 (99% agreement), and AD gave a kappa 1 (100% agreement).

### Patient and public involvement

A patient and public involvement group made up of OA patients (University of Bristol), reviewed the plans for this analysis at an early stage^35^. They supported the overall research aim and they emphasised the importance to focus on hip pain. The results of this work will be shared with the same group as well as the wider public and patient communities via social media and our university press teams.

### Statistical analysis

The demographic data are given as mean and range for continuous variables and binary variables are given as counts and frequency. Due to the clear differences in cam prevalence between the sexes, sex stratified analyses were conducted alongside combined sex models. We examined associations between hip morphologies and the presence of rHOA and its constituent features (osteophytes and JSN), using logistic regression. The results are presented as odds ratios (OR) with 95% confidence intervals (CI), comparing those having each morphology with the remainder. A sensitivity analysis was done comparing pincer morphology and AD with all rHOA based outcomes using logistic regression with a reference group including those with a LCEA ≥25° & <45° as both ends of the LCEA spectrum have been associated with rHOA (Supplementary Results). Logistic regression was also used to examine relationships between morphology and hip pain. Directed acyclic graphs informed the *a priori* selection of covariates for the adjusted model, namely age, height, weight and ethnicity, with sex also added to the adjusted combined sex models. Sensitivity analyses were performed with rHOA grade ≥2 as the outcome. All statistical analyses used Stata version 15 (StataCorp, College Station, TX, USA).

## Results

### Population characteristics

7,000 UKB participants with a left hip DXA were initially selected, 193 were excluded (due to poor image quality or removal of consent) leaving 6,807 individuals (mean age: 62.7 years) in the final analysis. The sample comprised 3425 [50.3%] females and 3382 [49.7%] males. 1489 [21.9%] participants, 581 [17.2%] males and 908 [26.5%] females, had a self-reported diagnosis of OA (no joint locations were specified in the question) and 594 [8.7%] participants, 219 [6.5%] males and 375 [11.0%] females, reported hip pain for more than 3 months.

### DXA-derived hip shape characteristics

AA was greater in males [mean: 51.6° (range: 35.8–106.2)] than females [44.2° (33.2–115.0)] and cam morphology, defined as AA ≥60°, was more frequently found in males [519 (15.4%)] than females [63 (1.8%)] (Table I). LCEA was similar in males [35.5° (7.9–61.8)] and females [35.2° (8.4–59.7)] with pincer morphology, defined as LCEA ≥45°, showing a similar prevalence in males [300 (8.9%)] and females [278 (8.1%)]. AD, defined as LCEA <25°, was slightly more common in females [238 (7.0%)] compared with males [188 (5.6%)].

**Table I:** Descriptive statistics for the UK Biobank sample used in this study

Demographics	Males	Females	Combined
	Mean [Range]	Mean [Range]	Mean [Range]
Age (years)	63.4 [45–80]	62.1 [46–79]	62.7 [45–80]
Weight (kg)	83.8 [50–160]	68.7 [36–155]	76.2 [36–160]
Height (cm)	177.0 [153–203]	163.3 [137–195]	170.1 [137–203]
Hip Pain	219 [6.5%]	375 [11.0%]	594 [8.7%]
*Ethnicity*	Prevalence [%]	Prevalence [%]	Prevalence [%]
White	3278 [97.0]	3321 [97.0]	6599 [97.0]
Asian	48 [1.4]	26 [0.8]	74 [1.1]
Black	23 [0.7]	20 [0.6]	43 [0.6]
Mixed heritage	13 [0.4]	21 [0.6]	34 [0.5]
Chinese	5 [0.2]	9 [0.3]	14 [0.2]
Unknown	15 [0.4]	28 [0.8]	43 [0.6]
*FAI and rHOA measures*	Prevalence [%]	Prevalence [%]	Prevalence [%]
Cam (AA ≥60°)	519 [15.4]	63 [1.8]	582 [8.6]
Pincer (LCEA ≥45°)	300 [8.9]	278 [8.1]	578 [8.5]
AD (LCEA <25°)	188 [5.6]	238 [7.0]	426 [6.3]
rHOA	245 [7.2]	108 [3.2]	353 [5.2]
Acetabular OP	485 [14.3]	345 [10.1]	830 [12.2]
Superior Femoral OP	291 [8.6]	143 [4.2]	434 [6.4]
Inferior Femoral OP	168 [5.0]	52 [1.5]	220 [3.2]
JSN	817 [24.2]	543 [15.9]	1360 [20]
rHOA grade ≥2	105 [3.1]	23 [0.7]	128 [1.9]
**Total Sample**	3382	3425	6807

### rHOA and its constituent features

Prevalent rHOA, defined as the presence of a grade ≥1 osteophyte combined with grade ≥1 JSN, was more frequent in males [245 (7.2%)] than females [108 (3.2%)] (Table I). JSN was more common in males [817 (24.2%)] than females [543 (15.9%)]. Osteophytes at one or more locations were more frequent in males [709 (21%)] than females [448 (13.1%)], as were osteophytes at single locations [acetabular: male 14.3% vs female 10.1%; superior femoral: male 8.6% vs female 4.2%; inferior femoral: male 5.0% vs female 1.5%].

### Cam vs rHOA and its constituent features

Cam morphology was associated with an increased risk of rHOA in males [OR: 3.24 (95% CI 2.44–4.30; Table II)], females [2.73 (1.07–6.94; Table III)], and males and females combined [4.08 (3.15–5.27; Supplementary Table 1)]. Similar associations were seen after adjustment for demographic covariates, namely age, height, weight and ethnicity, with sex added to the combined sex model. In addition, cam morphology was associated with JSN in unadjusted and adjusted analyses in males [1.53 (1.25–1.88) & 1.53 (1.24–1.88) respectively (Table II)], females [1.83 (1.03–3.25) & 1.75 (0.97–3.14) respectively (Table III)], and males and females combined [1.88 (1.56–2.27) & 1.56 (1.28–1.89) respectively (Supplementary Table 1)].

**Table II:** Results from logistic regressions examining the relationships between different hip morphologies, and rHOA, as well as grade ≥1 osteophytes and JSN in males. Unadjusted and adjusted results are shown in the form of odds ratios (OR), 95% confidence intervals (CI) and *p*-values (*P*). Adjusted models include age, height, weight and ethnicity. rHOA, radiographic hip osteoarthritis; OP, osteophyte; JSN, joint space narrowing

Males										
	rHOA		Acetabular OP		Superior Femoral OP		Inferior Femoral OP		JSN	
	OR [95% CI]	*P*	OR [95% CI]	*P*	OR [95% CI]	*P*	OR [95% CI]	*P*	OR [95% CI]	*P*
Unadjusted analysis										
Cam	3.24 [2.44–4.30]	3.47 × 10^−16^	1.89 [1.50–2.39]	1.04 × 10^−07^	1.94 [1.46–2.58]	4.61 × 10^−06^	4.77 [3.46–6.57]	1.47 × 10^−21^	1.53 [1.25–1.88]	4.88 × 10^−05^
Pincer	1.30 [0.85–1.97]	0.22	0.88 [0.62–1.25]	0.49	0.62 [0.37–1.02]	0.06	0.86 [0.48–1.53]	0.60	4.03 [3.16–5.13]	1.86 × 10^−29^
AD	0.87 [0.48–1.58]	0.64	1.34 [0.91–1.97]	0.13	1.06 [0.63–1.77]	0.83	1.86 [1.09–3.19]	0.02	0.28 [0.17–0.47]	1.30 × 10^−06^
Adjusted analysis										
Cam	3.20 [2.41–4.25]	9.24 × 10^−16^	1.87 [1.48–2.36]	2.02 × 10^−07^	1.94 [1.45–2.57]	5.74 × 10^−06^	4.75 [3.44–6.57]	3.13 × 10^−21^	1.53 [1.24–1.88]	6.02 × 10^−05^
Pincer	1.30 [0.85–1.98]	0.22	0.86 [0.61–1.23]	0.41	0.63 [0.38–1.05]	0.08	0.81 [0.45–1.45]	0.47	4.15 [3.25–5.30]	7.52 × 10^−30^
AD	0.89 [0.49–1.62]	0.70	1.41 [0.96–2.08]	0.08	1.07 [0.64–1.79]	0.79	1.95 [1.13–3.35]	0.02	0.28 [0.16–0.47]	1.30 × 10^−06^

**Table III:** Results from logistic regression examining the relationships between different hip morphologies, and rHOA, as well as grade ≥1 osteophytes and JSN in females. Unadjusted and adjusted results are shown in the form of odds ratios (OR), 95% confidence intervals (CI) and *p*-values (*P*). Adjusted models include age, height, weight and ethnicity. rHOA, radiographic hip osteoarthritis; OP, osteophyte; JSN, joint space narrowing

Females										
	rHOA		Acetabular OP		Superior Femoral OP		Inferior Femoral OP		JSN	
	OR [95% CI]	*P*	OR [95% CI]	*P*	OR [95% CI]	*P*	OR [95% CI]	*P*	OR [95% CI]	*P*
Unadjusted analysis										
Cam	2.73 [1.07–6.94]	0.04	1.12 [0.51–2.47]	0.78	2.01 [0.80–5.10]	0.14	10.97 [4.93–24.39]	4.24 × 10^−09^	1.83 [1.03–3.25]	0.04
Pincer	1.30 [0.69–2.45]	0.43	0.91 [0.60–1.39]	0.68	1.24 [0.70–2.18]	0.45	2.09 [0.97–4.48]	0.06	4.03 [3.10–5.24]	1.31 × 10^−25^
AD	0.64 [0.26–1.59]	0.34	1.15 [0.76–1.75]	0.50	0.68 [0.31–1.47]	0.33	1.12 [0.40–3.13]	0.83	0.31 [0.18–0.54]	3.43 × 10^−05^
Adjusted analysis										
Cam	2.47 [0.96–6.36]	0.06	0.99 [0.45–2.21]	0.99	1.83 [0.72–4.67]	0.20	10.07 [4.49–22.61]	2.13 × 10^−08^	1.75 [0.97–3.14]	0.06
Pincer	1.23 [0.65–2.33]	0.53	0.83 [0.54–1.26]	0.38	1.15 [0.65–2.03]	0.64	1.96 [0.91–4.23]	0.09	4.05 [3.10–5.3]	1.52 × 10^−24^
AD	0.72 [0.29–1.79]	0.48	1.37 [0.90–2.09]	0.15	0.75 [0.35–1.64]	0.48	1.28 [0.46–3.62]	0.64	0.34 [0.19–0.58]	1.10 × 10^−04^

**Fig. 2:**
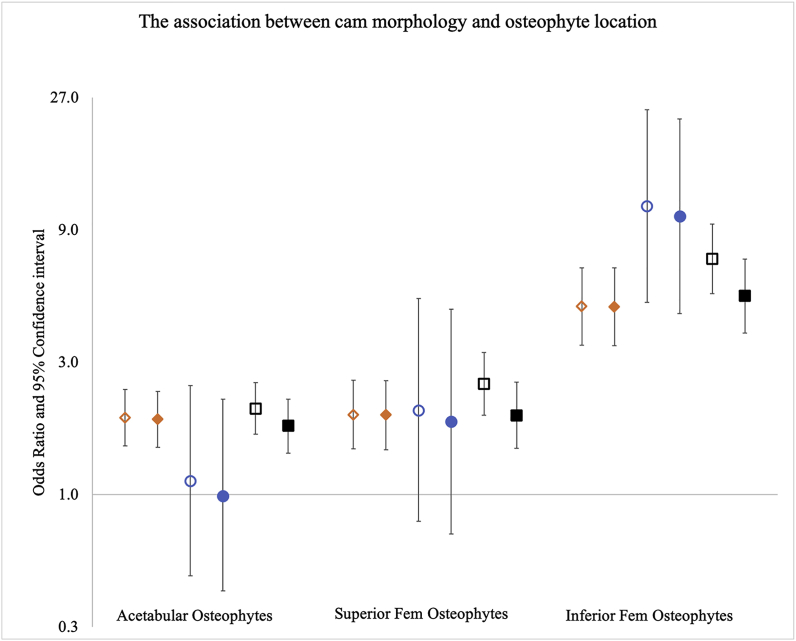
Logistic regression results are shown for the associations between cam morphology and osteophyte presence at three locations: acetabular, superior femoral, and inferior femoral head. Odds ratios are plotted with 95% confidence intervals either side. Results are presented as different models, diamonds represent the male only model (n = 3382), circles represent the female only model (n = 3425) and squares represent the combined sex model (n = 6807). Unadjusted results are shown by hollow shapes and results adjusted for age, height, weight and ethnicity are shown by filled shapes. The adjusted combined sex model also has sex as an additional covariate. *Y*-axis is natural log based.

In males, cam morphology was strongly associated with osteophytes at all locations in both unadjusted [acetabular osteophyte: 1.89 (1.50–2.39); superior osteophyte: 1.94 (1.46–2.58); inferior osteophyte 4.77 (3.46–6.57)] and adjusted analyses [acetabular osteophyte: 1.87 (1.48–2.36); superior osteophyte: 1.94 (1.45–2.57); inferior osteophyte 4.75 (3.44–6.57)] (Fig. 2 & Table II). In females, cam morphology was only associated with inferior femoral osteophytes, with equivalent results in unadjusted and adjusted analyses [10.97 (4.93–24.39) & 10.07 (4.49–22.62) respectively] (Fig. 2 & Table III). In sex-combined analyses, cam morphology was associated with osteophytes at all locations (Fig. 2 & Supplementary Table 1).

In sensitivity analyses based on rHOA grade ≥2, associations equivalent to those above were seen in males (Supplementary Table 2) and females (Supplementary Table 3), with the exception that these showed little evidence of an association between cam morphology and grade ≥2 inferior femoral osteophytes in females.

### Pincer and AD vs rHOA and its constituent features

There was little evidence of association between pincer morphology and rHOA, in males, females, or males and females combined (Table II, Table III, Supplementary Table 1). In contrast, pincer morphology showed strong associations with JSN in males [4.03 (3.16–5.130], females [4.03 (3.10–5.24)], and males and females combined [4.00 (3.36–4.77)], with equivalent findings after adjustment. Pincer morphology was unrelated to the presence of osteophytes. AD was unrelated to rHOA or osteophytes in males, females, or males and females combined (Table II and 3, Supplementary Table 1). In contrast, AD was negatively associated with JSN in males [0.28 (0.17–0.47)], females [0.31 (0.18–0.54)], and males and females combined [0.29 (0.20–0.42)], with equivalent findings after adjustment (Table II and 3, Supplementary Table 1). A sensitivity analysis was conducted for pincer morphology and AD, comparing their associations with rHOA based outcomes with those of a reference group which included those without AD and pincer morphology, yielding similar results (Supplementary Table 4).

### Morphological measures vs hip pain

Cam morphology was associated with hip pain in males, in both unadjusted and adjusted analyses [1.51 (1.08–2.12) and 1.48 (1.05–2.09) respectively] (Table IV). In further analyses, this association was partially attenuated by additional adjustment for the presence of osteophytes [adjusted OR for the presence of acetabular 1.43 (1.01–2.01), superior 1.42 (1.01–2.00), inferior 1.30 (0.91–1.85) osteophytes and all osteophytes combined 1.27 (0.89–1.81)]. In contrast, cam morphology was unrelated to hip pain in females, or males and females combined apart from in the adjusted model (Supplementary Table 5). There was no evidence of association between pincer or AD and hip pain, in males, females, or males and females combined (Table IV and Supplementary Tables 4 and 5).

**Table IV:** Results from logistic regression examining the relationship between hip shape morphologies and hip pain. The results are sex stratified and presented as odd ratios (OR), 95% confidence intervals (CI) and *p*-values (*P*). The adjusted models included age, height, weight and ethnicity

	Males				Females			
	Unadjusted		Adjusted		Unadjusted		Adjusted	
	OR [95% CI]	*P*	OR [95% CI]	*P*	OR [95% CI]	*P*	OR [95% CI]	*P*
Cam	1.51 [1.08–2.12]	0.02	1.48 [1.05–2.09]	0.02	1.19 [0.56–2.51]	0.65	1.11 [0.52–2.37]	0.78
Pincer	0.97 [0.60–1.58]	0.92	0.89 [0.54–1.45]	0.63	0.98 [0.66–1.46]	0.93	0.95 [0.63–1.41]	0.78
AD	1.17 [0.67–2.06]	0.58	1.27 [0.72–2.24]	0.41	1.24 [0.83–1.83]	0.29	1.32 [0.88–1.96]	0.18

## Discussion

In a large cross-sectional study of 6,807 individuals, we found that cam morphology was associated with an increased risk of prevalent hip OA, as reflected by rHOA and self-reported hip pain. In contrast, neither pincer morphology nor AD were related to either rHOA or hip pain, although they were associated with a greater and lower risk of JSN respectively. To further understand the relationship between cam morphology and hip OA, we explored the relationship between cam morphology and osteophyte distribution. Cam morphology was associated most strongly with inferior femoral head osteophytes, rather than those at the superior-lateral femoral head and acetabulum. In addition, the association between cam morphology and hip pain was partially attenuated by adjusting for the presence of inferior femoral osteophytes. This suggests that a mechanism involving the inferior femoral head contributes to the relationship between cam morphology and hip pain.

This is the first study to use DXA scans to define FAI-related morphologies with AA and LCEA. Comparison between DXA-derived AA [males: mean 51.6° (range 35.8–106.2); females: 44.2° (33.2–115.0)] and LCEA [males: 35.5°, (7.9–61.8); females: 35.2° (8.4–59.7)] from our study with comparative studies which used x-rays to derive AA [males: 52.6° (30–108); females: 45°, 26–92)] and LCEA [males: 34.4° (8–62); females: 35.3° (6–67)] show similar population level statistics^7^^,^^36^. Our findings are also consistent with results from previous population studies showing that cam morphology is associated with rHOA^5^^,^^6^. However, in contrast to the presented results, previous large population studies found no relationship between cam and hip pain^7^. In our study, cam morphology was predominantly a male characteristic, and although cam was associated with hip pain in males, a similar relationship was not seen in females, possibly due to a lack of power. These findings are consistent with previous work suggesting that cam is much less likely to occur in females and therefore cannot explain the majority of female hip OA or hip pain^34^. It may be that different thresholds for cam morphology based on AA are required in males and females, to account for sex differences in hip shape but further research is needed^10^^,^^36^.

Further, our findings are consistent with previous studies which found that pincer morphology is not associated with rHOA or hip pain^5^^,^^19^, and provide further evidence against an important role of pincer-type FAI in the development of hip OA. Though pincer morphology was unrelated to rHOA or osteophytes, it was associated with an increased risk of JSN. This could be a true relationship, but we are cautious of this conclusion as analysis of the site of maximal JSN showed this tended to be more lateral. This might represent an artefact related to 2-dimensional imaging creating the appearance of a narrowed joint space in the presence of acetabular over coverage which could represent a limitation when examining this outcome against an acetabulum-based hip morphology.

The lack of association between AD and hip OA in our study is in keeping with a previous study by Gosvig *et al.*^7^, but contrary to other previous studies^5^^,^^6^, in particular a systematic review which reported that longitudinal studies found acetabular under coverage associated with OA progression^37^. This maybe because acetabular coverage can mimic osteophytes and vice versa, despite high resolution images being inspected individually it can still be difficult to discriminate the two features thus potentially preventing cross-sectional studies from detecting associations between AD and rHOA. Direct comparisons between studies are difficult because of the different LCEA cut-offs used to define AD, along with differences in the imaging modalities used and outcomes employed. For example, Saberi Hosnijeh *et al.* used a more stringent threshold of LCEA (<20°) (compared to <25° in the present study) and reported associations between AD and total hip replacement (THR) as opposed to rHOA or hip pain.

Whilst any mechanistic links cannot be reliably determined in the context of this cross-sectional analysis, it is possible that the relationship between cam morphology and rHOA is causal, such that pre-existing cam morphology causes aberrant biomechanical forces which in turn lead to osteophyte formation. Since the strongest associations were observed between cam morphology and inferior femoral osteophytes, as opposed to superior femoral and acetabular osteophytes, this suggest aberrant biomechanical forces are present throughout the joint. Our study did not show a predisposition for osteophytes at the site of impingement, i.e., acetabular or superior femoral head osteophytes. This aligns with a previous study that found cam-type hip shape modes obtained from statistical shape modelling derived from DXA scans were associated with osteophytes both superiorly and inferiorly on the acetabulum and femoral head measured on x-rays taken 5 years later^38^. Other authors have suggested inferior femoral head osteophytes to be a marker of hip instability but further work is needed to understand how cam morphology might contribute to this^39^.

The association between cam morphology and hip pain which we observed may partly be mediated by osteophyte formation, particularly inferior osteophytes, adjustment for which led to partial attenuation of this relationship. Although not a formal mediation analysis this indicates that osteophyte formation may mediate the relationship between cam morphology and hip pain. This is consistent with findings from our recent study based on the same DXA images, where we found osteophytes at different locations to be independently associated with hip pain^25^. This view is also in agreement with several other emerging lines of evidence that osteophytes are an important source of pain in hip OA^40^, ^41^, ^42^.

This represents the largest population study to date of relationships between hip morphology and hip OA, which was made feasible by the development of automated means of deriving AA and LCEA on hip DXA scans. However, although well suited for derivation of hip morphology^38^ and rHOA^22^, use of DXA scans has some inherent limitations. For example, when deriving LCEA, since only one hip is visualised per scan, it was not possible to adjust for pelvic tilt as performed when deriving equivalent measures from radiographs^19^. Another limitation arises from examining only left hips when the hip pain measure used in our study was not side specific. The latter reduces precision, although this would likely bias our results towards the null rather than inducing false associations. Another limitation is the cross-sectional nature of our study. For example, it is possible that spurious associations may be introduced between hip morphology and rHOA, if measures such as AA and LCEA incorporate osteophytes because it is difficult to identify the true contour of the bone and as already mentioned we cannot comment on causality of any observations seen. Unfortunately, our study does not include measures of subchondral sclerosis or cysts which are well recognised constituents of rHOA again decreasing the precision of our measurement of rHOA. Additionally, DXA scans are done supine rather than weight bearing which could theoretically increase mJSW. However, a comparison between JSW on weight bearing and non-weight bearing hip x-rays found only a minimal change in JSW (0.1 mm mean difference) in those who already had JSN^43^ and OARSI clinical trial guidance suggests supine hip x-rays are acceptable for assessing rHOA^44^. Finally, our study is based on 2-dimensional imaging which limits our ability to detect differences in hip morphology in planes better visualised on 3-dimensional imaging^45^. Of note is that a recent study comparing x-rays with CT scans showed similar sensitivity and specificity between the two modalities when defining cam and pincer morphology^46^.

In conclusion, using novel methods developed and applied to high resolution DXA images from a large cross-sectional study, we found that cam morphology is associated with hip OA, as reflected by rHOA and self-reported hip pain. These associations were strongest in men, in whom cam morphology was much more common than in women. We found associations between cam morphology and osteophytes to be located throughout the joint with the strongest relationship with those at the inferior femoral head. Further work is needed to understand the biomechanical consequences of cam morphology underlying the pattern of osteophytes with which this is associated, as a prelude to developing tailored strategies for reducing OA progression.

## Author contributions

All authors have made significant contributions to the conception and design of this study, the acquisition of data, its analysis and interpretation. All authors helped draft the article before approving the final version of this manuscript. Dr B Faber (ben.faber@bristol.ac.uk) takes responsibility for the integrity of the work in its entirety.

## Conflcit of interest

TC & CL have a patent Image processing apparatus and method for fitting a deformable shape model to an image using random forest regression voting. This is licensed with royalties to Audax, and to Optasia Medical. NH reports consultancy fees and honoraria from UCB, Amgen, Kyowa Kirin, Thornton Ross, Consilient.

## Role of the funding source

BGF is supported by a 10.13039/501100000265Medical Research Council (MRC) clinical research training fellowship (MR/S021280/1). RE, MF, FS are supported, and this work is funded by a Wellcome Trust collaborative award (reference number 209233). CL was funded by the MRC, UK (MR/S00405X/1). NCH acknowledges support from the MRC and NIHR Southampton Biomedical Research Centre, 10.13039/501100000739University of Southampton and 10.13039/100010417University Hospital Southampton. BGF, MF, GDS & JHT work in the MRC Integrative Epidemiology Unit at the University of Bristol, which is supported by the MRC (MC_UU_00,011/1). No funders had any role in the study design, collection, analysis and interpretation of data; in the writing of the manuscript; and in the decision to submit the manuscript for publication.
