# Supplementary material for: Cam morphology but neither acetabular dysplasia nor pincer morphology is associated with osteophytosis throughout the hip: findings from a cross-sectional study in UK Biobank
Source: Osteoarthritis Cartilage. 2021 Nov;29(11):1521–9. doi: 10.1016/j.joca.2021.08.002 (PMC8547486; doi:10.1016/j.joca.2021.08.002)
Supplement: Multimedia component 1 [file mmc1.docx]

**Supplementary Material**

Supplementary Table 1. Results from logistic regressions examining the relationships between different hip morphologies, and rHOA, as well as grade ≥1 osteophytes and JSN in a combined sex analysis. Unadjusted and adjusted results are shown in the form of odds ratios (OR), 95% confidence intervals (CI) and p-values (*P*). Adjusted models include age, sex, height, weight and ethnicity. rHOA, radiographic hip osteoarthritis; OP, osteophyte; JSN, joint space narrowing.

| Combined sex analysis | | | | | | | | | | |
| --- | --- | --- | --- | --- | --- | --- | --- | --- | --- | --- |
| Unadjusted analysis | | | | | | | | | | |
|  | rHOA | | Acetabular OP | | Superior Femoral OP | | Inferior Femoral OP | | JSN | |
|  | OR [95% CI] | *P* | OR [95% CI] | *P* | OR [95% CI] | *P* | OR [95% CI] | *P* | OR [95% CI] | *P* |
| Cam | 4.08 [3.15-5.27] | 9.32 x 10^-27^ | 2.04 [1.65-2.53] | 7.49 x 10^-11^ | 2.51 [1.93-3.26] | 6.89 x 10^-12^ | 7.08 [5.30-9.45] | 2.80 x 10^-40^ | 1.88 [1.56-2.27] | 4.04 x 10^-11^ |
| Pincer | 1.32 [0.93-1.87] | 0.12 | 0.90 [0.69-1.18] | 0.47 | 0.82 [0.56-1.19] | 0.30 | 1.14 [0.72-1.81] | 0.57 | 4.00 [3.36-4.77] | 9.70 x 10^-54^ |
| AD | 0.75 [0.45-1.23] | 0.25 | 1.22 [0.92-1.62] | 0.17 | 0.87 [0.57-1.33] | 0.52 | 1.52 [0.95-2.44] | 0.08 | 0.29 [0.20-0.42] | 8.39 x 10^-11^ |
| Adjusted analysis | | | | | | | | | | |
|  | rHOA | | Acetabular OP | | Superior Femoral OP | | Inferior Femoral OP | | JSN | |
|  | OR [95% CI] | *P* | OR [95% CI] | *P* | OR [95% CI] | *P* | OR [95% CI] | *P* | OR [95% CI] | *P* |
| Cam | 3.14 [2.40-4.12] | 1.10 x 10^-16^ | 1.77 [1.41-2.21] | 6.20 x 10^-07^ | 1.93 [1.47-2.54] | 2.24 x 10^-06^ | 5.20 [3.83-7.06] | 4.97 x 10^-26^ | 1.56 [1.28-1.89] | 9.13 x 10^-06^ |
| Pincer | 1.27 [0.90-1.81] | 0.18 | 0.84 [0.64-1.11] | 0.22 | 0.80 [0.55-1.17] | 0.25 | 1.05 [0.66-1.66] | 0.85 | 4.08 [3.41-4.89] | 1.2 x 10^-52^ |
| AD | 0.82 [0.50-1.36] | 0.45 | 1.38 [1.04-1.83] | 0.03 | 0.94 [0.61-1.44] | 0.78 | 1.75 [1.09-2.83] | 0.02 | 0.30 [0.21-0.44] | 4.61 x 10^-10^ |

| Males (grade ≥2) | | | | | | | | | | |
| --- | --- | --- | --- | --- | --- | --- | --- | --- | --- | --- |
| Unadjusted analysis | | | | | | | | | | |
|  | rHOA | | Acetabular OP | | Superior Femoral OP | | Inferior Femoral OP | | JSN | |
|  | OR [95% CI] | *P* | OR [95% CI] | *P* | OR [95% CI] | *P* | OR [95% CI] | *P* | OR [95% CI] | *P* |
| Cam | 3.91 [2.62-5.85] | 3.00 x 10^-11^ | 2.00 [1.51-2.65] | 1.50 x 10^-06^ | 1.90 [1.33-2.71] | 3.82 x 10^-04^ | 5.99 [3.47-10.35] | 1.42 x 10^-10^ | 1.64 [1.24-2.16] | 4.81 x 10^-04^ |
| Pincer | 1.75 [1.00-3.06] | 0.05 | 1.09 [0.72-1.64] | 0.68 | 0.74 [0.40-1.34] | 0.32 | 1.07 [0.42-2.71] | 0.88 | 4.51 [3.41-5.98] | 9.22 x 10^-26^ |
| AD | 1.42 [0.68-2.96] | 0.35 | 1.19 [0.73-1.95] | 0.48 | 1.25 [0.68-2.29] | 0.47 | 2.65 [1.18-5.94] | 0.02 | 0.44 [0.22-0.86] | 1.72 x 10^-02^ |
| Adjusted analysis | | | | | | | | | | |
|  | rHOA | | Acetabular OP | | Superior Femoral OP | | Inferior Femoral OP | | JSN | |
|  | OR [95% CI] | *P* | OR [95% CI] | *P* | OR [95% CI] | *P* | OR [95% CI] | *P* | OR [95% CI] | *P* |
| Cam | 3.85 [2.57-5.77] | 7.13 x 10^-11^ | 1.96 [1.48-2.61] | 2.98 x 10^-06^ | 1.86 [1.31-2.66] | 6.01 x 10^-04^ | 5.81 [3.36-10.05] | 3.20 x 10^-10^ | 1.62 [1.23-2.14] | 6.75 x 10^-04^ |
| Pincer | 1.62 [0.92-2.85] | 0.09 | 1.04 [0.69-1.58] | 0.83 | 0.76 [0.42-1.39] | 0.38 | 1.01 [0.40-2.57] | 0.98 | 4.50 [3.38-5.98] | 5.99 x 10^-25^ |
| AD | 1.59 [0.76-3.35] | 0.22 | 1.27 [0.77-2.07] | 0.35 | 1.25 [0.68-2.30] | 0.47 | 2.89 [1.28-6.54] | 0.02 | 0.45 [0.23-0.89] | 0.02 |

Supplementary Table 2. Results from logistic regression examining the relationships between different hip morphologies, and rHOA grade ≥2, as well as grade ≥2 osteophytes and JSN in males. Unadjusted and adjusted results are shown in the form of odds ratios (OR), 95% confidence intervals (CI) and p-values (*P*). Adjusted models include age, height, weight and ethnicity. rHOA, radiographic hip osteoarthritis; OP, osteophyte; JSN, joint space narrowing.

Supplementary Table 3. Results from logistic regression examining the relationships between different hip morphologies, and rHOA grade ≥2, as well as grade ≥2 osteophytes and JSN in females. Unadjusted and adjusted results are shown in the form of odds ratios (OR), 95% confidence intervals (CI) and p-values (*P*). Adjusted models include age, height, weight and ethnicity. rHOA, radiographic hip osteoarthritis; OP, osteophyte; JSN, joint space narrowing.

| Females (grade ≥2) | | | | | | | | | | |
| --- | --- | --- | --- | --- | --- | --- | --- | --- | --- | --- |
| Unadjusted analysis | | | | | | | | | | |
|  | rHOA | | Acetabular OP | | Superior Femoral OP | | Inferior Femoral OP | | JSN | |
|  | OR [95% CI] | *P* | OR [95% CI] | *P* | OR [95% CI] | *P* | OR [95% CI] | *P* | OR [95% CI] | *P* |
| Cam | 2.45 [0.32-18.45] | 0.38 | 1.36 [0.49-3.78] | 0.56 | 1.42 [0.34-5.9] | 0.63 | 2.70 [0.36-20.40] | 0.34 | 2.58 [1.09-6.08] | 3.09 x 10^-02^ |
| Pincer | 1.71 [0.5-5.78] | 0.39 | 0.97 [0.54-1.74] | 0.93 | 1.49 [0.74-3.02] | 0.27 | 4.61 [1.77-11.97] | 1.72 x 10^-03^ | 5.02 [3.39-7.44] | 8.59 x 10^-16^ |
| AD | 1.28 [0.3-5.48] | 0.74 | 0.77 [0.39-1.53] | 0.45 | 0.53 [0.17-1.69] | 0.28 | 0.67 [0.09-5.00] | 0.69 | 0.39 [0.14-1.06] | 6.55 x 10^-02^ |
| Adjusted analysis | | | | | | | | | | |
|  | rHOA | | Acetabular OP | | Superior Femoral OP | | Inferior Femoral OP | | JSN | |
|  | OR [95% CI] | *P* | OR [95% CI] | *P* | OR [95% CI] | *P* | OR [95% CI] | *P* | OR [95% CI] | *P* |
| Cam | 2.12 [0.28-16.07] | 0.47 | 1.15 [0.41-3.23] | 0.80 | 1.18 [0.28-4.96] | 0.82 | 1.98 [0.26-15.27] | 0.51 | 2.42 [1.01-5.78] | 0.05 |
| Pincer | 1.59 [0.47-5.42] | 0.46 | 0.85 [0.47-1.53] | 0.59 | 1.31 [0.64-2.66] | 0.46 | 3.95 [1.51-10.37] | 5.26 x 10^-03^ | 5.01 [3.34-7.5] | 6.03 x 10^-15^ |
| AD | 1.51 [0.35-6.53] | 0.58 | 0.97 [0.48-1.93] | 0.92 | 0.64 [0.20-2.07] | 0.46 | 0.92 [0.12-6.97] | 0.94 | 0.44 [0.16-1.2] | 0.11 |

Supplementary Table 4. Results from a sensitivity analysis using logistic regressions to examine the relationships between different hip morphologies, and rHOA, as well as grade ≥1 osteophytes, JSN and hip pain in a combined sex analysis where the reference group have neither pincer nor acetabular dysplasia. Unadjusted and adjusted results are shown in the form of odds ratios (OR), 95% confidence intervals (CI) and p-values (*P*). Adjusted models include age, sex, height, weight and ethnicity. rHOA, radiographic hip osteoarthritis; OP, osteophyte; JSN, joint space narrowing.

| Combined sex analysis | | | | | | | | | | | | | |
| --- | --- | --- | --- | --- | --- | --- | --- | --- | --- | --- | --- | --- | --- |
| Unadjusted analysis | | | | | | | | | | | | | |
|  | rHOA | | Acetabular OP | | Superior Femoral OP | | Inferior Femoral OP | | JSN | | Hip Pain | | |
|  | OR [95% CI] | *P* | OR [95% CI] | *P* | OR [95% CI] | *P* | OR [95% CI] | *P* | OR [95% CI] | *P* | OR [95% CI] | *P* |  |
| Pincer | 1.3 [0.92-1.84] | 0.14 | 0.92 [0.7-1.2] | 0.54 | 0.82 [0.56-1.19] | 0.29 | 1.18 [0.75-1.88] | 0.47 | 3.8 [3.18-4.53] | 1.20 x 10^-49^ | 0.98 [0.72-1.34] | 0.91 |  |
| AD | 0.77 [0.47-1.26] | 0.30 | 1.21 [0.91-1.61] | 0.18 | 0.86 [0.56-1.31] | 0.48 | 1.55 [0.96-2.48] | 0.07 | 0.34 [0.23-0.49] | 1.63 x 10^-08^ | 1.25 [0.91-1.73] | 0.30 |  |
| Adjusted analysis | | | | | | | | | | |  |  | |
|  | rHOA | | Acetabular OP | | Superior Femoral OP | | Inferior Femoral OP | | JSN | | Hip Pain | | |
|  | OR [95% CI] | *P* | OR [95% CI] | *P* | OR [95% CI] | *P* | OR [95% CI] | *P* | OR [95% CI] | *P* | OR [95% CI] | *P* |  |
| Pincer | 1.27 [0.89-1.8] | 0.19 | 0.86 [0.65-1.13] | 0.28 | 0.79 [0.54-1.16] | 0.23 | 1.1 [0.69-1.75] | 0.69 | 3.9 [3.26-4.68] | 3.00 x 10^-49^ | 0.95 [0.69-1.29] | 0.23 |  |
| AD | 0.84 [0.51-1.39] | 0.50 | 1.36 [1.03-1.82] | 0.03 | 0.93 [0.6-1.42] | 0.73 | 1.76 [1.09-2.85] | 0.02 | 0.35 [0.24-0.51] | 5.44 x 10^-08^ | 1.3 [0.94-1.8] | 0.11 |  |

| Combined sex analysis | | | | |
| --- | --- | --- | --- | --- |
|  | Unadjusted | | Adjusted | |
|  | OR [95% CI] | *P* | OR [95% CI] | *P* |
| Cam | 1.08 [0.8-1.44] | 0.62 | 1.40 [1.02-1.9] | 0.03 |
| Pincer | 0.97 [0.71-1.31] | 0.82 | 0.92 [0.68-1.26] | 0.62 |
| AD | 1.25 [0.91-1.73] | 0.17 | 1.31 [0.95-1.81] | 0.10 |

Supplementary Table 5 Results from logistic regression examining the relationships between different hip morphologies, and hip pain in a combined sex analysis. Unadjusted and adjusted results are shown in the form of odds ratios (OR), 95% confidence intervals (CI) and p-values (*P*). Adjusted models include age, sex, height, weight and ethnicity.
